# Does Presence of a Mid-Ocean Ridge Enhance Biomass and Biodiversity?

**DOI:** 10.1371/journal.pone.0061550

**Published:** 2013-05-02

**Authors:** Imants G. Priede, Odd Aksel Bergstad, Peter I. Miller, Michael Vecchione, Andrey Gebruk, Tone Falkenhaug, David S. M. Billett, Jessica Craig, Andrew C. Dale, Mark A. Shields, Gavin H. Tilstone, Tracey T. Sutton, Andrew J. Gooday, Mark E. Inall, Daniel O. B. Jones, Victor Martinez-Vicente, Gui M. Menezes, Tomasz Niedzielski, Þorsteinn Sigurðsson, Nina Rothe, Antonina Rogacheva, Claudia H. S. Alt, Timothy Brand, Richard Abell, Andrew S. Brierley, Nicola J. Cousins, Deborah Crockard, A. Rus Hoelzel, Åge Høines, Tom B. Letessier, Jane F. Read, Tracy Shimmield, Martin J. Cox, John K. Galbraith, John D. M. Gordon, Tammy Horton, Francis Neat, Pascal Lorance

**Affiliations:** 1 Oceanlab, Institute of Biological and Environmental Sciences, University of Aberdeen, Aberdeen, United Kingdom; 2 Institute of Marine Research, Flødevigen, His, Norway; 3 Remote Sensing Group, Plymouth Marine Laboratory, Plymouth, United Kingdom; 4 National Oceanic and Atmospheric Administration/National Marine Fisheries Service, National Systematics Laboratory, National Museum of Natural History, Smithsonian Institution, Washington, District of Columbia, United States of America; 5 P.P. Shirshov Institute of Oceanology, Russian Academy of Sciences, Moscow, Russia; 6 National Oceanography Centre, Southampton, United Kingdom; 7 Scottish Association for Marine Science, Scottish Marine Institute, Oban, United Kingdom; 8 Virginia Institute of Marine Science, College of William & Mary, Gloucester Point, Virginia, United States of America; 9 Department of Oceanography and Fisheries, University of the Azores, Horta, Faial, Portugal; 10 Department of Geoinformatics and Cartography, Institute of Geography and Regional Development, University of Wrocław, Wrocław, Poland; 11 Marine Research Institute, Reykjavik, Iceland; 12 Scottish Oceans Institute, University of St. Andrews, United Kingdom; 13 School of Biological and Biomedical Sciences, Durham University, Durham, United Kingdom; 14 Institute of Marine Research, Bergen, Norway; 15 Centre for Marine Futures, Oceans Institute, University of Western Australia, Perth, Western Australia, Australia; 16 Southern Ocean Ecosystem Change Department, Australian Antarctic Division, Kingston, Tasmania, Australia; 17 Northeast Fisheries Science Center, National Oceanic and Atmospheric Administration, Woods Hole, Massachusetts, United States of America; 18 Marine Laboratory, Marine Scotland Science, Aberdeen, United Kingdom; 19 Institut Français de Recherche Pour L'exploitation De La Mer, Nantes, France; Heriot-Watt University, United Kingdom

## Abstract

In contrast to generally sparse biological communities in open-ocean settings, seamounts and ridges are perceived as areas of elevated productivity and biodiversity capable of supporting commercial fisheries. We investigated the origin of this apparent biological enhancement over a segment of the North Mid-Atlantic Ridge (MAR) using sonar, corers, trawls, traps, and a remotely operated vehicle to survey habitat, biomass, and biodiversity. Satellite remote sensing provided information on flow patterns, thermal fronts, and primary production, while sediment traps measured export flux during 2007–2010. The MAR, 3,704,404 km^2^ in area, accounts for 44.7% lower bathyal habitat (800–3500 m depth) in the North Atlantic and is dominated by fine soft sediment substrate (95% of area) on a series of flat terraces with intervening slopes either side of the ridge axis contributing to habitat heterogeneity. The MAR fauna comprises mainly species known from continental margins with no evidence of greater biodiversity. Primary production and export flux over the MAR were not enhanced compared with a nearby reference station over the Porcupine Abyssal Plain. Biomasses of benthic macrofauna and megafauna were similar to global averages at the same depths totalling an estimated 258.9 kt C over the entire lower bathyal north MAR. A hypothetical flat plain at 3500 m depth in place of the MAR would contain 85.6 kt C, implying an increase of 173.3 kt C attributable to the presence of the Ridge. This is approximately equal to 167 kt C of estimated pelagic biomass displaced by the volume of the MAR. There is no enhancement of biological productivity over the MAR; oceanic bathypelagic species are replaced by benthic fauna otherwise unable to survive in the mid ocean. We propose that globally sea floor elevation has no effect on deep sea biomass; pelagic plus benthic biomass is constant within a given surface productivity regime.

## Introduction

The Mid-Atlantic Ridge (MAR) was described a century ago as the most striking feature of the Atlantic Ocean dividing the ocean into eastern and western deep basins [1]. By the 1950s sonar surveys [2] had revealed the structure of the MAR with a tectonically active central rift valley bounded by elevated flanks on either side, sloping down to the abyssal plains [3]. This forms part of the global mid-ocean ridge system occupying 33% of the total ocean floor that plays a major role in plate tectonics as the site of formation of new earth's crust [4]. Whilst the geological function of the mid-ocean ridge system is well known, its biological significance remains uncertain. Abundant chemosynthetically-supported life is found around hydrothermal vents that occur along ridge axes [5]. However despite their ubiquity, locally high biomass [6] and productivity [7], vent fields are small and sparsely distributed [8] so can only make a minor contribution to mid-ocean biological productivity. Downward export of organic carbon from photosynthesis in surface layers of the ocean is the dominant source of secondary biological productivity over mid-ocean ridge systems.

Mid-ocean shallows such as ridges and seamounts have attracted attention as areas of high fisheries productivity [9], [10], [11] and biodiversity [12], [13]. Generally, the most biologically productive regions of the oceans are coastal shallow seas with high incident solar radiation and rapid recycling of nutrients from the sea floor augmented by terrestrial inputs. In the open ocean, nutrient concentrations in surface layers are restricted and a significant fraction of surface primary production is exported downward into the ocean interior, gradually attenuated with depth, supporting deep-sea life throughout the water column and on the abyssal sea floor [14]. Export production can vary with time and is reported to be 50–80% of the primary production during episodic blooms or in high productivity areas, but much lower (5–10%) outside of these periods due to recycling and re-mineralisation of organic matter in the photic zone [15]. Benthic biomass decreases with increasing depth and distance from the continents so that at abyssal depths it is<1% of the values in coastal waters [16]. Thus in mid ocean in the absence of a ridge - relatively low surface productivity would support a sparse abyssal fauna at >4 km depth. The presence of a mid-ocean ridge with a truncated water columns disrupts this general pattern potentially creating regions of high biomass that may arise from topographic influences on water circulation [17] upwelling nutrient-rich deep water as well as concentrating biomass over summits creating mid ocean regions of high productivity. Sea surface temperature fronts that are typically areas of elevated primary production [18], may account for enhanced production if associated with ridge topography. Elevation of the sea floor is likely to provide additional habitat for slope-dwelling bathyal fauna. Such species cannot otherwise survive in mid ocean owing to their adaptation to restricted species-specific depth ranges. Biodiversity maxima tend to occur at mid-slope depths around the ocean margins [16], if this trend were reflected at similar depths in mid ocean this would greatly enhance biodiversity there. Mid-ocean shallows may thus provide stepping stones for trans-oceanic dispersal of bathyal species. Conversely the ridge may act as a barrier to movement of abyssal species between the two halves of the ocean. Finally, there may be sufficient isolation of bathyal fauna on the ridge to allow development of endemic species confined to the ridge system, further enhancing oceanic biodiversity.

There is concern that deep water biogenic habitats such as corals and sponge fields on mid-ocean ridges are vulnerable to damage from fishing and other anthropogenic activity. Despite great scientific uncertainty, high seas Marine Protected Areas (MPAs) have been established over large areas of the MAR [19] to conserve these habitats. Globally the mid-ocean ridge system is recognised as a large scale ecosystem with extensive areas of lower bathyal habitat defined as depths of 800–3500 m [20]. Although seamounts of similar depths have been proposed to be hotspots of biodiversity and biological productivity these paradigms have been questioned [21]. The present study is concerned with elucidating the potential multiple effects of the presence of a mid-ocean ridge system on oceanic biology.

**Figure 1:**
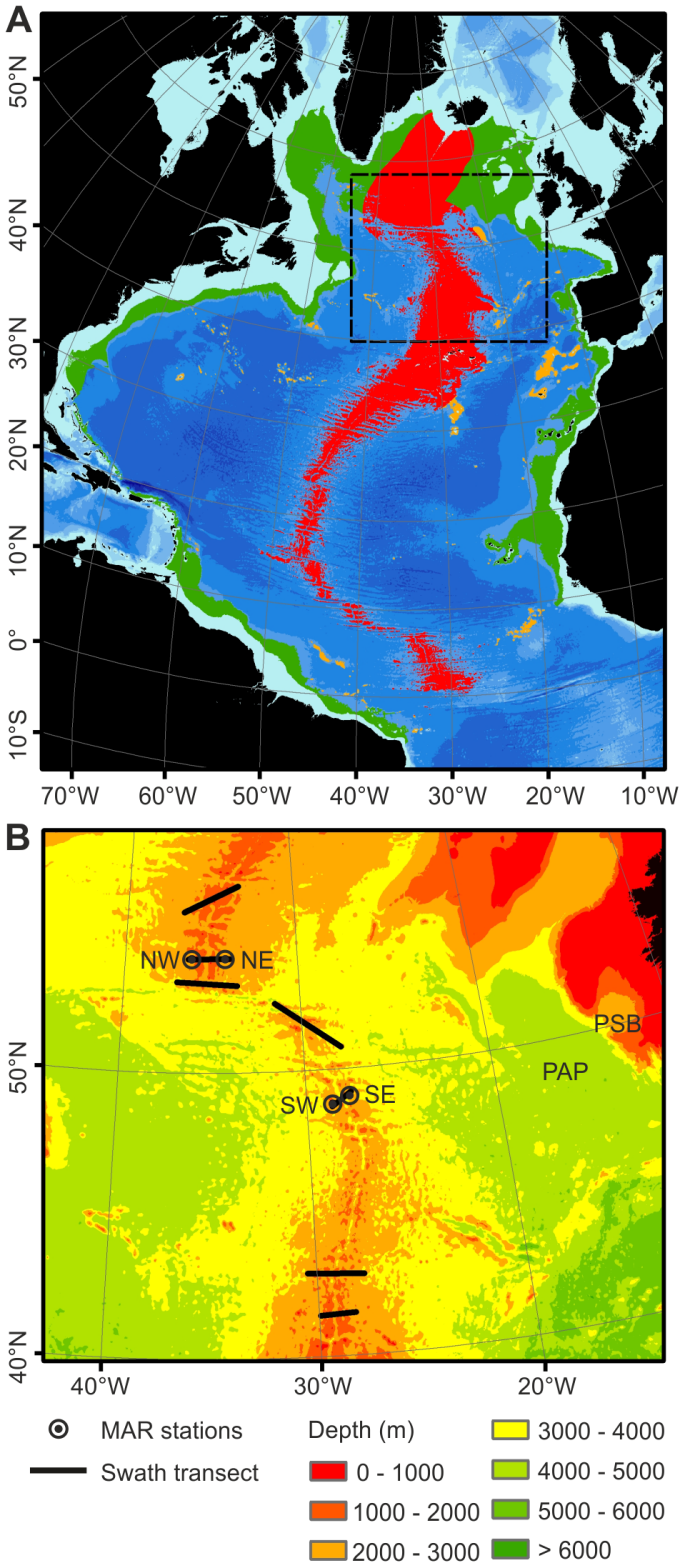
Analysis of lower bathyal area of the Atlantic Ocean. (A) The North Atlantic basin showing the lower bathyal zones (depths 800–3500 m) on the MAR (red), continental margins (green) and non-contiguous seamounts (orange). Dashed rectangle – area shown in Figure 1B. (B)NE, NW, SE, SW, - MAR stations at 2500 m depth with instrumented sediment trap moorings 2007–2010 and sites of detailed surveys. Swath bathymetry survey tracks. PAP - Porcupine Abyssal Plain PSB - Porcupine Seabight.

The present study has been conducted over an extensive segment of northern Mid-Atlantic Ridge (MAR), focussing on a region between the Azores and Iceland (Figure 1A) around the Charlie-Gibbs Fracture Zone (CGFZ). The CGFZ is a major discontinuity in the MAR at 53°N (Figure 1B) coinciding with the location of the sub-polar front (SPF) which delineates the boundary between Subarctic Intermediate Water (9–10°C) at the surface to the north and North Atlantic Central water (15–16°C, summer temperatures) to the south [22].

## Materials and Methods

For the purposes of large-scale analysis we have considered the entire North Atlantic basin deeper than 800 m (Figure 1A) north of a line between Natal, Brazil and Bolama, Guinea-Bissau using 30-second gridded global bathymetry data [23](corresponding to 926 m resolution in latitude). For detailed studies we sampled an area between the Azores and Iceland centred on the Charlie-Gibbs Fracture Zone (Figure 1A,B). In order to characterise the sea floor habitat, sonar swath bathymetry data were collected during voyages of the FRV *GO Sars* in 2004 (Kongsberg Simrad EM 300) [24], and RRS *James Cook* in 2007 (Kongsberg Simrad EM120). Data were viewed in real time on board the vessels using OLEX software but were post processed on shore with MB system V5 (www.mbari.org/data/mbsystem) or equivalent software for seven segments traversing the ridge (Figure 1B). Maps with resolution<100 m were generated and areas occupied by 3 slope categories calculated on an equal area projection: Flat plains (slope <5°), gentle slopes (5–30°) and steep slopes (>30°). Based on these data, four stations were established; NE (54°00.05′N 34°10.61′W), NW (53°59.33′N 36°07.39′W), SE (49°01.92′N 27°40.82′W) and SW (48°46.80′N 28°38.43′) spaced either side of the MAR axis (Figure 1B). The moorings were placed at 2500 m depth and sampling was conducted at depths from 2056 to 2762 m around the stations. In 2010, video line transects were done using the Remotely Operated Vehicle (ROV) *Isis*. As a laser pointer passed in a straight line over the sea floor the benthic habitat type, soft sediment or hard rock, was scored at 1 cm resolution using HD video camera at 2 m altitude above the sea floor. A total of 24 km were sampled in this way with 500 m long transects placed at random within the three slope categories. A mooring was placed at each station, with sediment traps 100 m and 1000 m above bottom, plus other instrumentation measured downward flux of organic matter and water flow over the ridge from July 2007 to June 2010. Voyages of the *RRS James Cook* serviced the moorings, surveyed and sampled in 2007, 2009 and 2010. *RRS Discovery* serviced the moorings in 2008 (see File S1). Cruise reports can be downloaded at http://www.oceanlab.abdn.ac.uk/ecomar/cruises.php. All data from these cruises and cruise reports are also archived and can be accessed from the British Oceanographic Data Centre: http://www.bodc.ac.uk.

Since all the sampling locations were in high seas areas beyond any national jurisdiction no specific permits were required for the described field studies.

However the work followed the “Code of conduct for responsible marine research in the deep seas and high seas of the OSPAR maritime area” [25].

Satellite remote sensing data were used to evaluate frequency of fronts and primary production over the MAR and adjacent regions. Thermal fronts were investigated using 8-day composite front maps [26] derived from daily merged microwave and infrared sea surface temperature data from 2006 to 2011, and then aggregated to indicate regions where strong fronts are most frequently observed. Primary production was estimated using a wavelength resolving model [27], [28] using mean monthly 9 km NASA SeaWiFS OC4v4 Chla and Pathfinder v2009 AVHRR SST data to generate mean monthly satellite maps of PP from 1997 to 2010. The satellite estimates of primary production are accurate to 20% in the Atlantic Ocean [29] and were verified by *in situ* incubation of samples on board ship. Advection of surface particles to the sediment traps was calculated based on altimeter-estimated surface currents and settlement velocities covering phytoplankton size range of 0.2–20 µm. Particles were found to advect from a 70 km radius over the NW and NE moorings and a 700 × 400 km ellipse over the SW and SE moorings. Primary and new production values were calculated from cloud free pixels over these areas. Missing data (28%) were estimated from interpolation using a Gaussian fit on the production climatology (r^2^ = 0.99) and annual production was calculated from the March-September mean for 2007–2010. Mass and organic carbon fluxes from the time-series sediment traps (McLane research Laboratories, Inc. MA, USA) at the 4 MAR stations were estimated using JGOFS (Joint Global Ocean Flux Study) protocols. http://usjgofs.whoi.edu/protocols_rpt_19.html

Two proxies were used to investigate patterns of pelagic biomass distribution above the MAR, acoustic surveys and bioluminescence. The Reykjanes Ridge section of the MAR and the adjacent Irminger Sea have been surveyed in June and July of each year (1996–2009) down to a depth of 850 m by an international fisheries acoustic survey for the pelagic redfish (*Sebastes mentella*). All non-redfish features on the echograms were designated as deep-scattering layer. Composition was verified by pelagic net tows. For depths from 500 m to the sea floor, data from vertical profiles of counts of bioluminescent organisms impacting on a fast-descending mesh screen were abstracted from surveys over the MAR [30], Porcupine Abyssal Plain (PAP) and Porcupine Seabight (PSB) (Figure 1B) [31].

Quantitative sampling of benthic fauna was done using a multiple corer (10 cm diameter core tubes) for macrofauna (see File S1[S2]) and an otter trawl (see File S1 [S4]) for megafauna and fishes. In addition, samples were collected by baited traps and in 2010 by ROV *Isis* equipped with manipulators, cores, grabs, suction samplers and a suite of cameras. Sampling was focussed on areas around the four instrumented stations (Fig 1B). Sampling over wider latitudinal (41°22′N to 60°18′N) and depth (607 to 3465 m) ranges during the voyage of the RV *GO Sars* in 2004 provided further data for assessment of species occurrences on the MAR [24].

## Results

### Bathymetry and benthic habitat

Using 30-second gridded global bathymetry data [32] we have estimated the area of sea floor at lower bathyal depths (800–3500 m) in the North Atlantic (Figure 1*A*). These habitats cover a total of 8,109,116 km^2^ with the MAR being the largest single area accounting for almost half of total area (Table 1).

**Table 1:** Areas of Lower Bathyal Habitat in the North Atlantic Ocean.

	Area (km^2^)	Percentage of Total
Mid-Atlantic Ridge (MAR)	3,704,404	45.68
Eastern Continental Slopes	1,793,261	22.11
Western Continental Slopes	2,297,983	28.34
Eastern Seamounts	222,467	2.74
Western Seamounts	91,001	1.12
TOTAL	8,109,116	100.00

**Figure 2:**
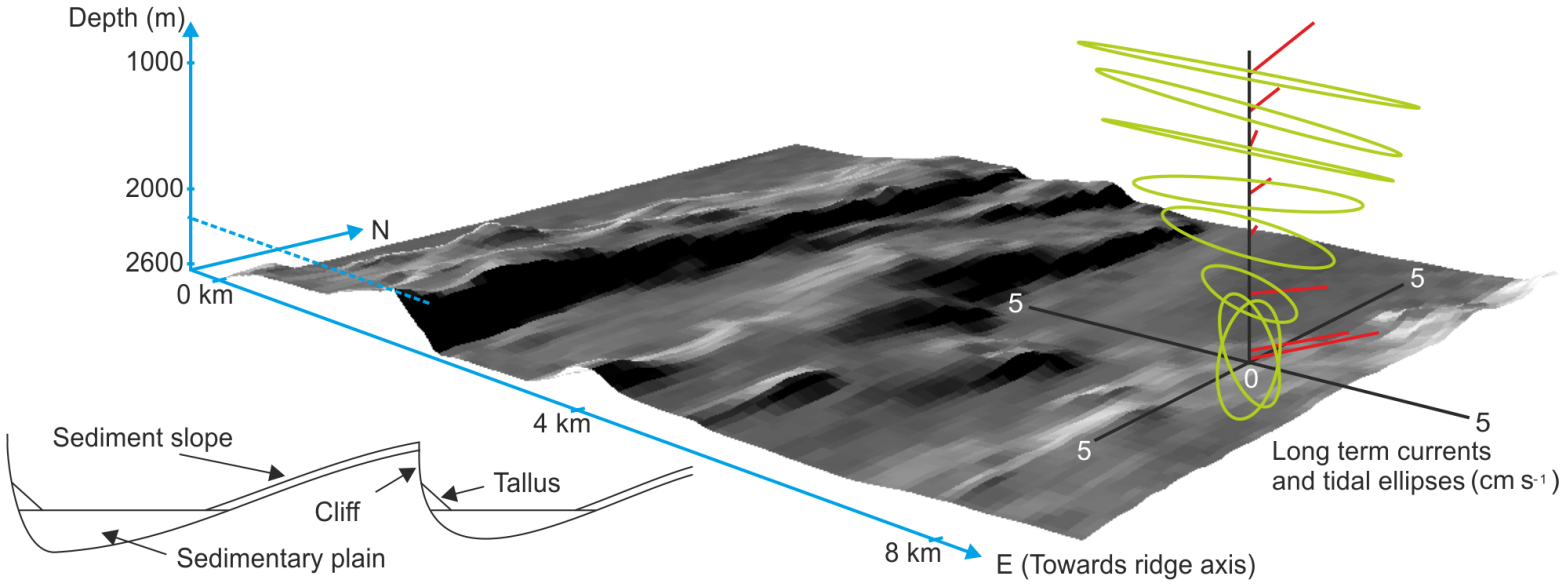
Sea floor topography and flow on the flanks of the MAR. (a) 3D projection from swath bathymetry surveys around the NW MAR station, vertical and horizontal scales the same. (b) Location of the NW mooring (base 2500 m depth) and flow at different heights above the sea floor. Red vectors - long term mean velocity. Green - ellipses of the dominant (M2) tidal constituent. (c) Diagrammatic cross section of a flank of the ridge showing the relationship between flat plains, gentle slopes and steep cliffs. The steep cliffs face towards the ridge axis.

**Figure 3:**
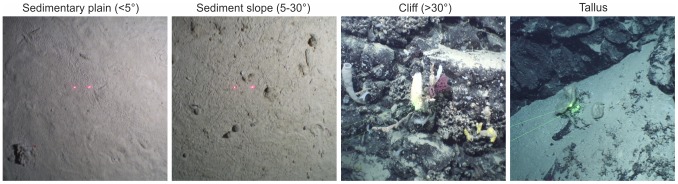
Sea floor images on the MAR. Optical ROV images of the sea floor in flat (<5°), gentle slope (5–30°) and steep (>30°) terrains on the MAR. Red or green laser spots are 10 cm apart.

Seven swath-bathymetry transects (Figure 1*B*) covering a total of 10093 km^2^ revealed that the flanks of the MAR are dominated by flat terraces separated by steep slopes, often with cliffs, parallel to and facing the ridge axis (Figure 2). Flat plains (slope <5°) comprise 37.7% of the area surveyed, gentle slopes (5–30°) 56.7% and steep slopes (>30°) 5.7%. High resolution video surveys by ROV *Isis* showed that sediment coverage was 100% on the flat plains, 98.4% on the gentle slopes and 33.1% sediment on the steep slopes. On the gentle slopes, sediment cover was interrupted by occasional rocky outcrops but on the steep slopes there were cliffs with bare rock on the vertical faces. A talus with a very unstable soft sediment slope was often present at the base of the cliff (Figure 3). Despite the presence of conspicuous rocky features we conclude that the predominant substrate on the MAR is fine soft sediment amounting to 95.3% of the lower bathyal area. Hard substrata in the form of rocky outcrops and cliff faces harbour a diverse assemblage of sessile fauna dominated by corals, sponges and crinoids [33], but the area is small.

Moored current meters showed near-bottom flow at sub-tidal frequencies to be strongly constrained to follow the typically north-south orientation of the topography (Figure 2b). Semidiurnal tidal flows, while largely cross-ridge at the surface, are topographically steered near the bed, combining with the sub-tidal flow to create semidiurnal pulsing or reversals of flow. When flow is forced to cross topography, internal bores, lee waves and enhanced turbulence occur [17]. Topography, substratum and flow variability are physical drivers of spatial and temporal habitat heterogeneity and on the MAR are comparable with or exceed those of ocean margins

### Fronts, primary production and export flux over the Mid-Atlantic Ridge

**Figure 4:**
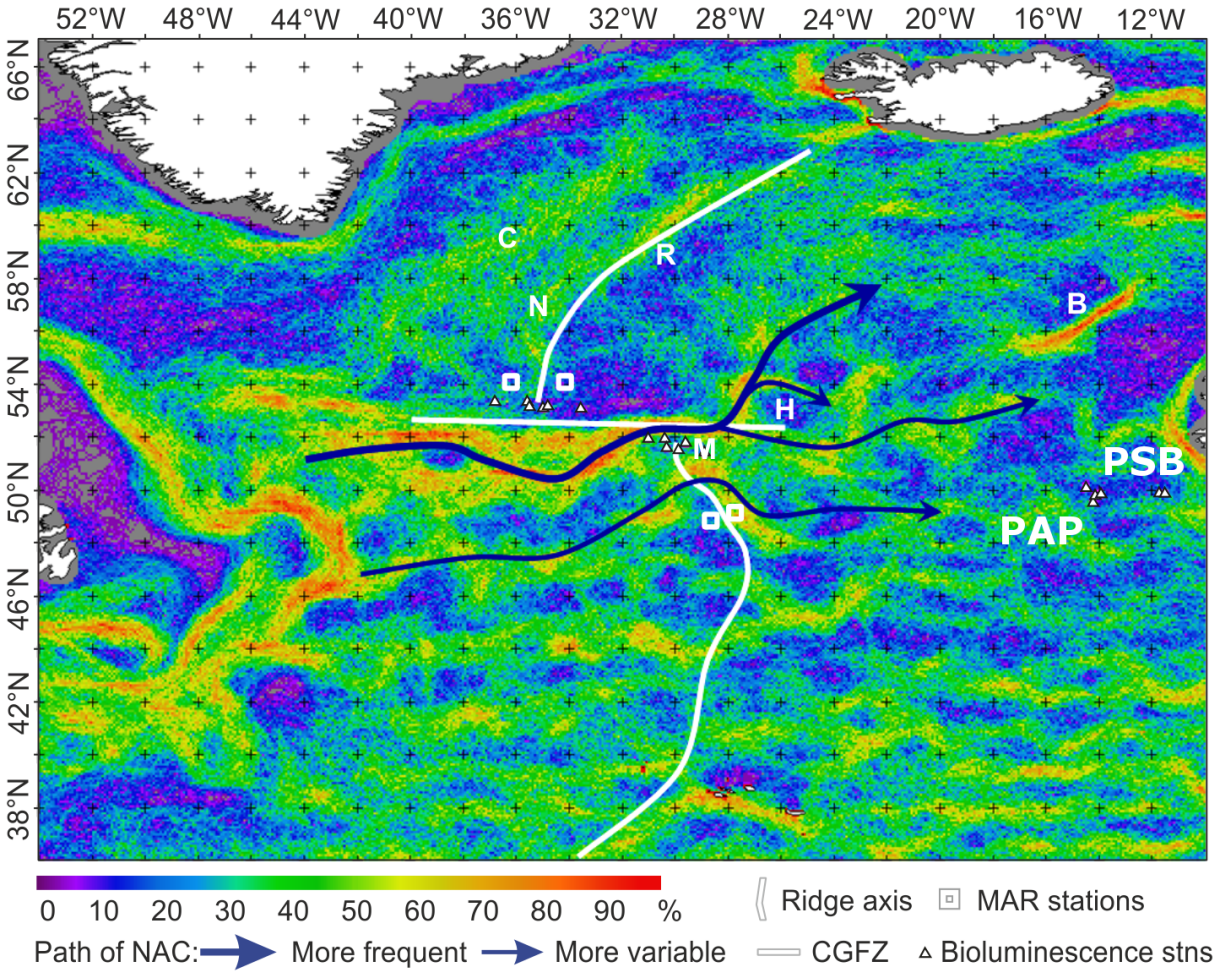
Remote sensing imagery of distribution of fronts. Seasonal oceanic front frequency map indicating the percentage of time a strong front was observed at each location during spring (Mar-May), derived from merged microwave and infrared SST (Sea Surface Temperature) data, 2007–2009. Blue arrows are inferred paths of branches of the North Atlantic Current (NAC) delineating the sub-polar front. Frontal features related to bathymetry are labelled: B - Rockall Bank, C - Iceland-Greenland branch of NAC, H - deeper hollow in sea bed, M - recurring meander, N - near to MAR, PAP – Porcupine Abyssal Plain, PSB – Porcupine Seabight, R – Reykjanes Ridge.

Satellite sea-surface temperature data reveals structure in the distribution of fronts (Figure 4), some of which can may reflect the influence of MAR topography and hence affect the distribution of plankton blooms [18]. The eastward flowing North Atlantic Current (NAC) tends to cross the MAR at deep fracture zones [22], particularly the CGFZ. Frequent thermal fronts delineate the northern edge of the sub-polar front (SPF) just south of the CGFZ. On the shallower Reykjanes Ridge section of the MAR (mean crest depth 987 m) fronts were detected near the ridge crest and to the west with no fronts to the east. South of the CGFZ the NAC comprises a continuous succession of energetic, long-lasting, slow moving eddies, confirmed by time-integrated thermal front analysis to be constrained to favour certain latitudinal bands (Figure 4). This is part of a large-scale pattern across the Atlantic Ocean with no evidence of an effect of the MAR south of 54°N. Remote sensing shows clear differences between the SE and SW stations with high frequency of fronts to the east but this cannot be attributed to a direct topographic effect.

Primary production values calculated from satellite remote sensing data for the period 1997–2009 were compared with a reference station on the Porcupine Abyssal Plain (PAP) east of the MAR. The mean primary production at the MAR stations (March-Sept) was 553 mgC.m^−2^d^−1^ or 202 gC.m^−2^ y^−1^, significantly lower than 625 mgC.m^−2^d^−1^ or 228 gC.m^−2^ y^−1^ over the same period at PAP (F_2,422_ = 10.84, P<0.0001) [14], [34], [35].

Sediment traps 1500 m below surface at the four MAR stations during 2007–2010 recorded a mean annual flux of 0.658 ±0.26 gC.m^−2^.y^−1^, lower than the 0.905 gC.m^−2^.y^−1^ recorded at 1000 m depth at PAP. Both at PAP and MAR a seasonal pattern was observed with high deposition, rich in phyto-detritus, during the summer months. We find no evidence for enhanced downward flux from the surface at the MAR. Aluminium concentrations in the sediment traps 100 m above sea floor indicate a contribution from resuspension of bottom sediment.

### Pelagic biota above the MAR

**Figure 5:**
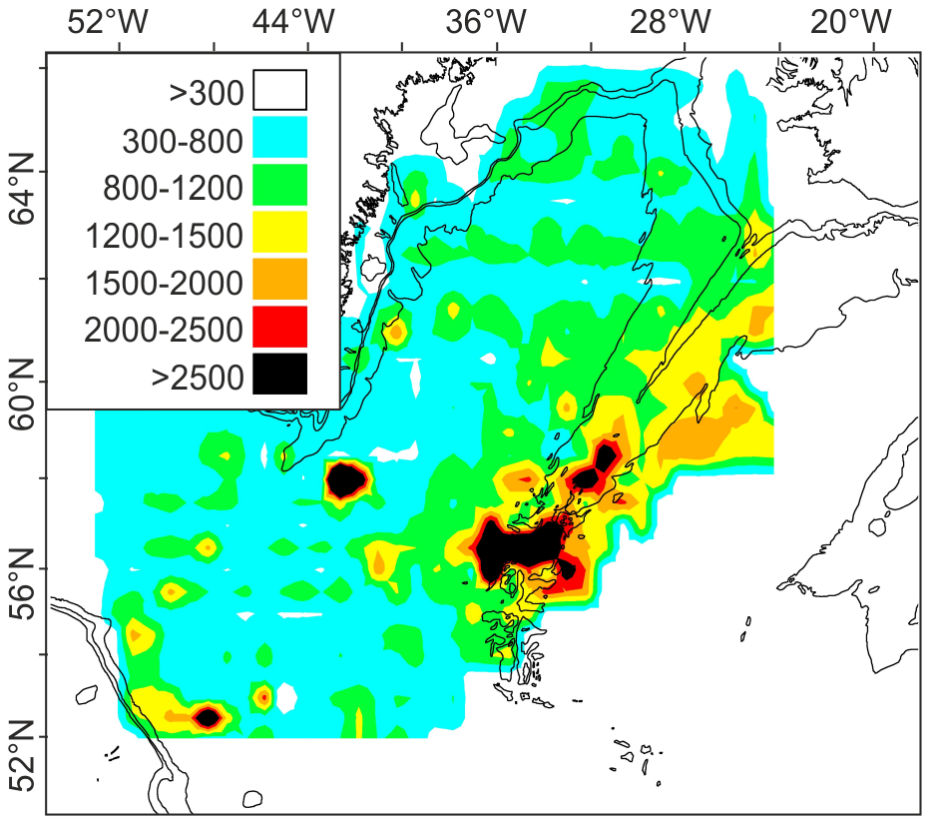
Spatial distribution of area backscatter. Area backscattering coefficient (m^2^ nautical mile^−2^), from the mesopelagic deep scattering layer. Recordings from multi-ship surveys using calibrated SIMRAD 38 kHz echosounders integrating down to 750 m depth (1000 m after 1999). Composite image of data from 1996–2009 during June–July.

Hydroacoustic surveys during 1996–2009 showed dense mesopelagic deep scattering layers at depths of 300–800 m (Figure 5). There was considerable seasonal and inter-annual variability but a consistent pattern emerged of high density near and across the crest of the Reykjanes Ridge during June and July which net samples showed comprised fishes and other micronekton.

**Figure 6:**
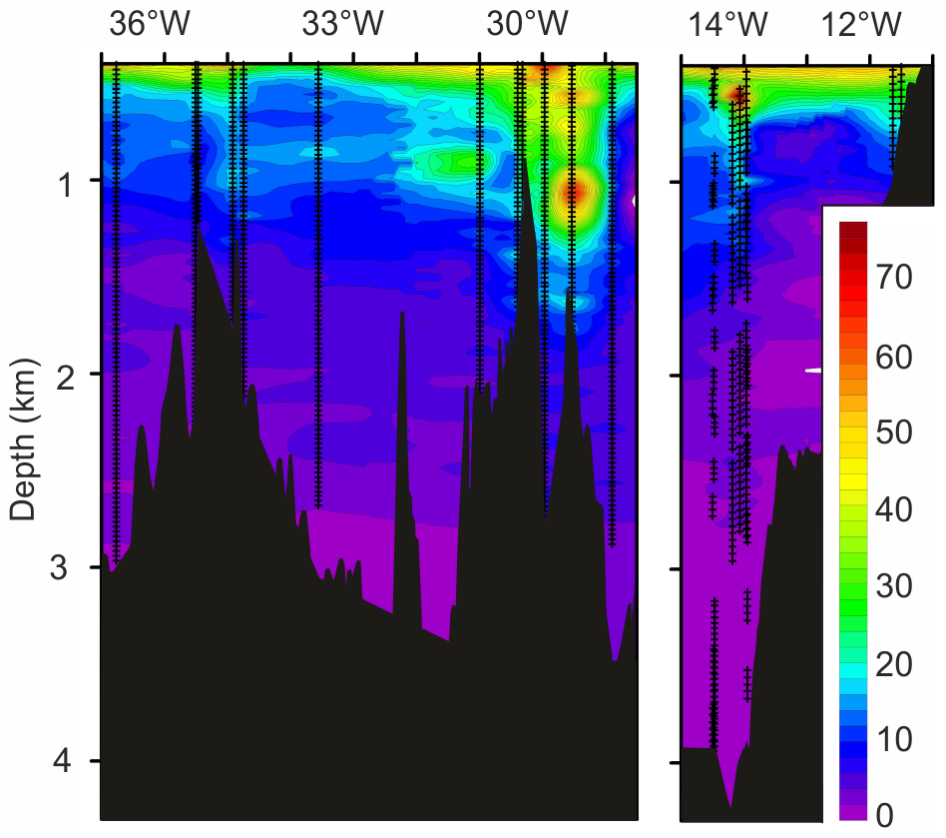
Vertical distribution of bioluminescent organisms. Abundance of organisms (number.m^−3^) between the MAR and the Porcupine Seabight from profiles at the triangle symbols in Figure 4. Black area is the sea floor.

Profiles of the abundance of bioluminescent animals showed no difference in mean abundance between the MAR and the North-East Atlantic margin (Figures 4 & 6) with 17.74±11.29 (SD) m^−3^ over the MAR and 17.13±12.28 m^−3^ over the PSB at depths 500–1500 m. High abundance was detected over the MAR in one profile at 29°32.07′W extending down to 1500 m depth. This was associated with a warm-water eddy and was not attributable to ridge topography (Figure 6).

### Benthic Biomass on the MAR

**Figure 7:**
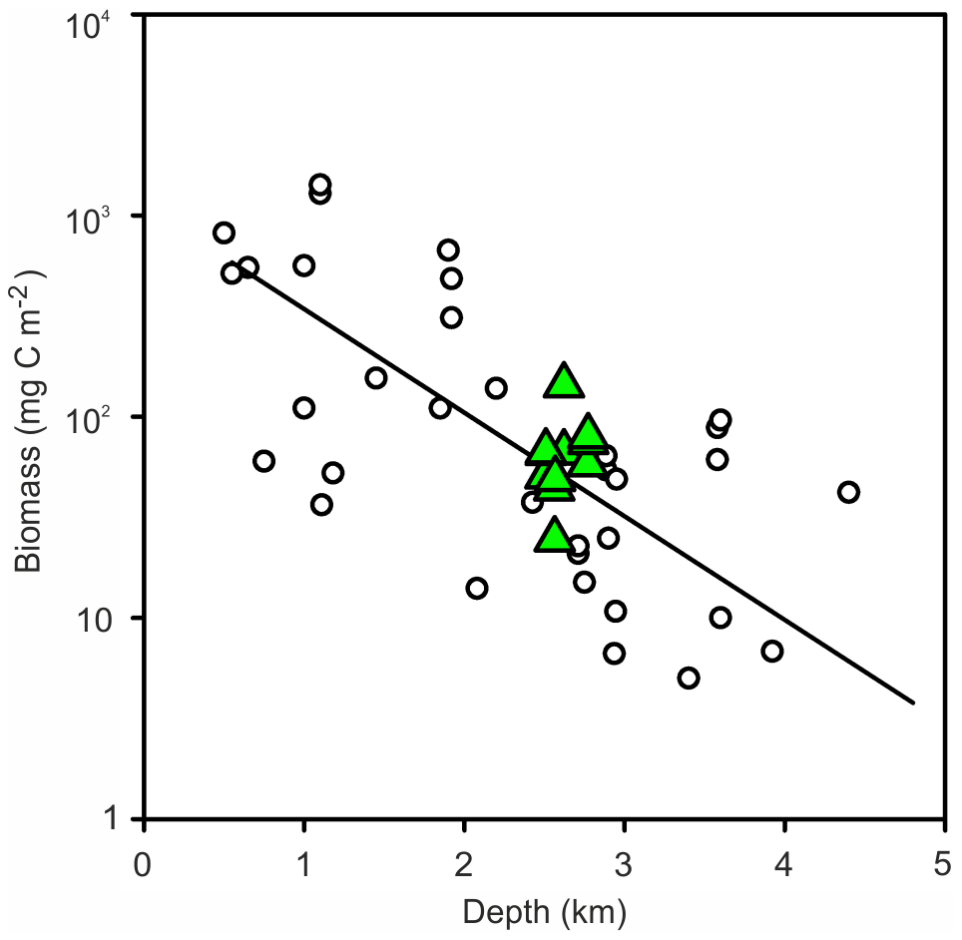
Macrofauna biomass. Data from sediment core samples at the four MAR stations (green triangles) compared with locations around the North Atlantic margin (open circles) and the CoML global trend line.

The mean macrofaunal biomass in sediment core samples from the four MAR stations was 56.10 (SD = 41.26, n = 11) mg C m^−2^. These values are comparable to samples from similar depths on the North Atlantic continental margins [36], [37] and straddle the trend line of the global predictive equation from the Census of Marine Life (CoML) Fresh Biomass Database for macrofauna [38] (Figure 7). The trend line predicts an expected biomass of 57.88 mg C m^−2^ at 2500 m depth, very close to our observed value.

**Figure 8:**
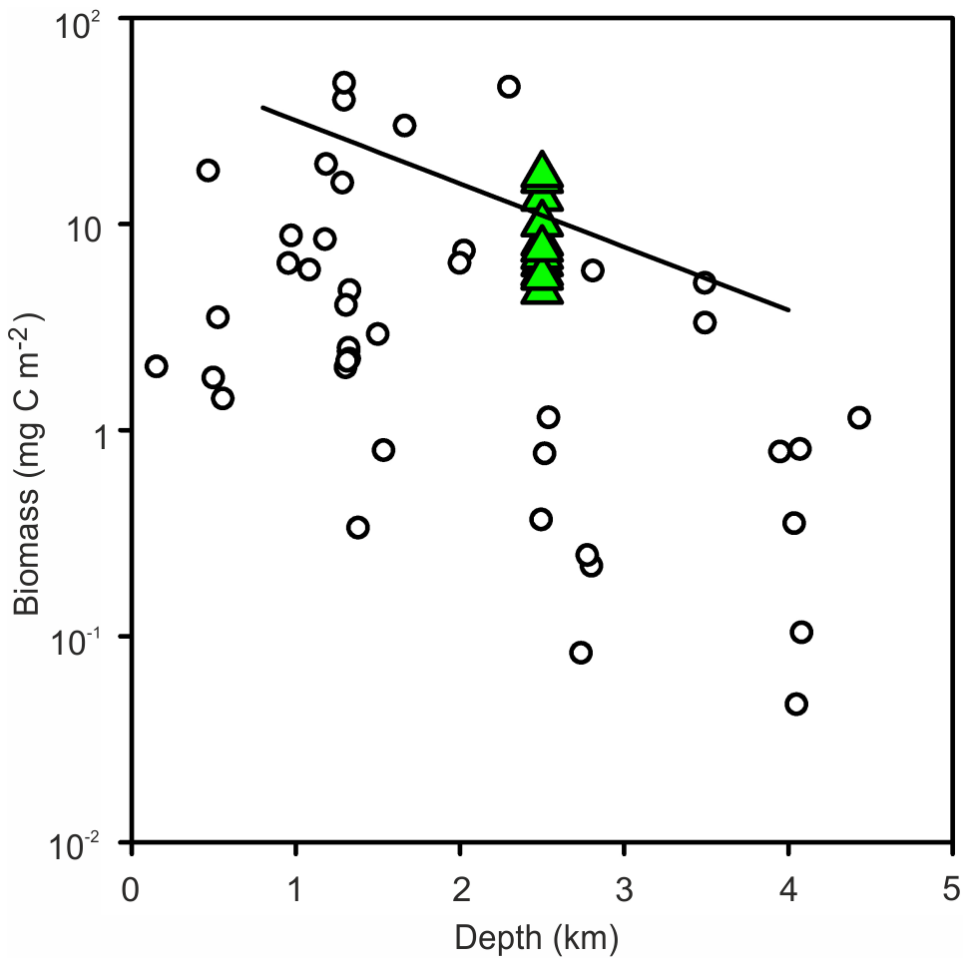
Benthic megafauna biomass. Data from trawl samples at the NW, NE and SW MAR stations (green triangles) compared with locations around the North Atlantic margin (open circles) and the CoML global trend line.

The mean benthic megafaunal biomass in demersal otter trawl (OTSB) catches at the NW, NE and SE MAR stations was 9.29 mg C m^−2^, SD = 4.44, n = 11 close to the predicted value of 11.02 mg C m^−2^ from the CoML Fresh Biomass Database for megafauna (Figure 8) with values lying around the trend line. However, the values are at the upper end of scatter of data from the same depths at the PSB on the North-East Atlantic margin, particularly for holothurians and to a lesser extent, echinoids and crustaceans.

**Figure 9:**
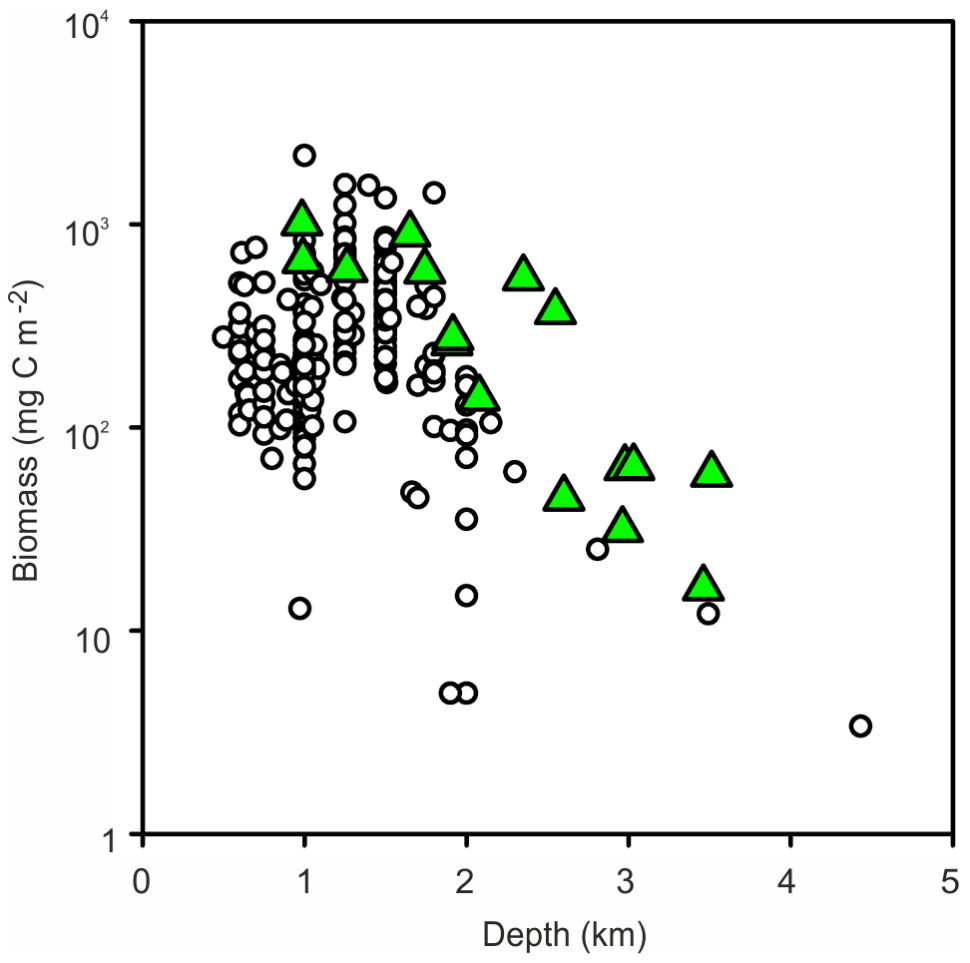
Demersal fish biomass. Data from trawl samples at the NW, NE and SW MAR stations (assuming C = 10% wet weight), (Green triangles) compared with locations around the North Atlantic margin (open circles).

Demersal fish biomass from the trawl samples at 2500 m depth was 0.90 kg 1000 m^−2^ (SD = 0.84, n = 11) on the MAR compared with 1.09 kg 1000 m^−2^ (SD = 0.44, n = 13) using the same gear (OTSB, [39]) at the same depths at the PSB. A wider analysis of trawl data from around the Atlantic Ocean and the MAR [40, S12, S13, S14, S15, S16, S17, S18], not including the present samples (Figure 9) reveals a general decline in total fish biomass with depth but a trend towards higher biomass on the MAR at depths greater than 2000 m.

### Total benthic biomass over the MAR and displacement of pelagic biomass

**Figure 10:**
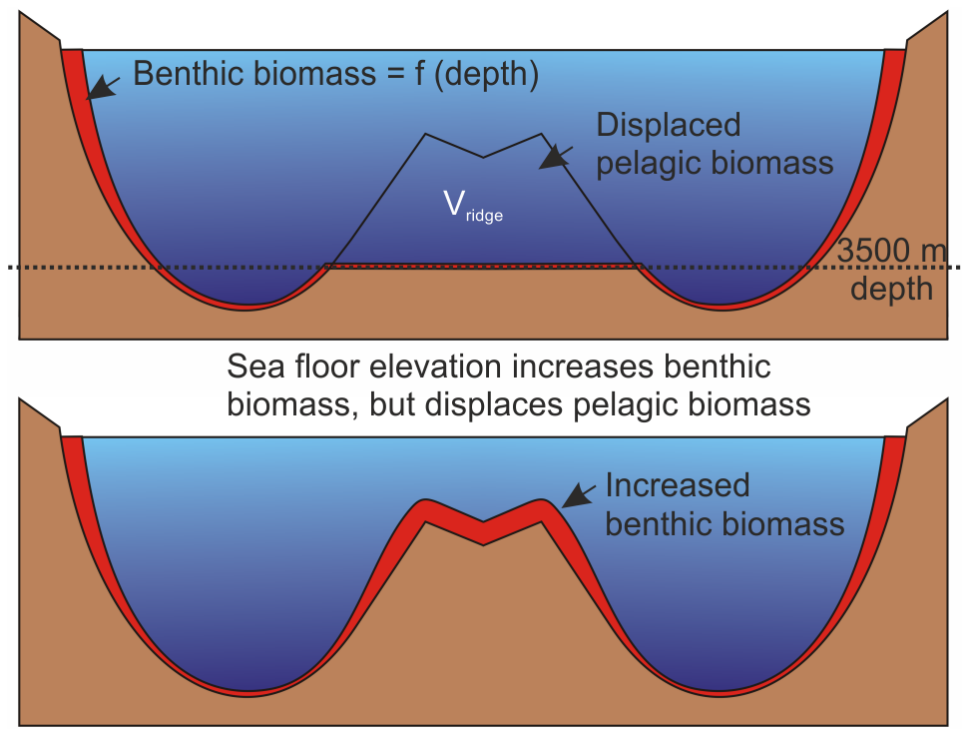
Effect of a ridge on mid-ocean biomass. Comparison of sections of a hypothetical ocean with the MAR truncated at the 3500 m depth horizon (upper panel) with the real ocean with a ridge present (lower panel). The thickness of the red section indicates the benthic biomass that decreases with depth. V_ridge_ is the volume of water, including pelagic biomass displaced by the ridge.

From the macrofauna, megafauna and fish data we conclude that the benthic biomass on the MAR does not deviate from global mean values for a given depth and hence the CoML global trend equations can be used to predict biomass on the MAR. We therefore developed a GIS model of the lower bathyal, based on GEBCO bathymetry and integrated the predicted biomass over the entire area of the MAR shown in Figure 1A. Predicted total macrofauna biomass is 220.6 kt C and megafauna 38.3 kt C, giving a total of 258.9 kt C for the benthic biomass over the entire lower bathyal habitat of the MAR. We compared this with the null hypothesis of a hypothetical Atlantic Ocean with no MAR (Figure 10). For a flat ocean floor 3500 m deep in place of the MAR the corresponding values are 65.5 and 20.1 = 85.6 kt C. This implies that elevation of the sea floor by the presence of the MAR increases Atlantic Ocean benthic biomass by 173.3 kt C. The COML random forest model [38] explains 81% of variance in benthic macrofauna biomass and standard deviation of global biomass estimates is ±44% of the mean.

Whilst elevation of the sea floor increases benthic biomass we calculate that the MAR also displaces 2.79 × 10^15^ m^3^ of pelagic habitat, thus removing biomass from the mid-ocean pelagic zone (Figure 10). For the North Atlantic, biomass of mesozooplankton at the BIOTRANS site 47°N 20°W between 4250 and 2250 m depth [41] has been estimated as 0.04 mg C.m^−3^. Taking into account smaller and larger plankton and nekton size classes we assume the pelagic biomass in water displaced by the ridge is likely to be greater. Assuming a mean pelagic biota biomass of 0.06 mg C.m^−3^ for the depth range 800–3500 m [S21, S22, S23, S24, S25, 42], displacement by the MAR reduces regional biomass by 167 kt C. Thus it appears that presence of the MAR increases benthic biomass by an amount approximately equal to the pelagic biomass lost so total biomass remains constant. Whilst the benthic biomass estimates are derived from a global data base of containing thousands of records [38] the deep pelagic is chronically under-sampled [43], so our comparison is based on relatively sparse pelagic biomass data.

### The relationship between pelagic and benthic biomass

Our observation of a neutral effect of the presence of the MAR on deep-sea biomass in the North Atlantic raises the question of whether globally, pelagic plus benthic biomass can be assumed to be constant (see File S1 and Figure S1).

Generally pelagic biomass decreases logarithmically with depth according to the relationship [42]:
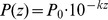
(1)Where P(z)  = pelagic biomass at depth z and P_0_ and k are constants.P_0_ is the intercept i.e. surface biomass density and k the decay constant or rate of decrease in biomass with depth. If pelagic plus benthic biomass at depths >800 m is assumed to be constant, then in water of depth H: 
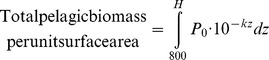
(2)


and



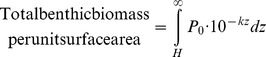
(3) (Figure 11).

**Figure 11:**
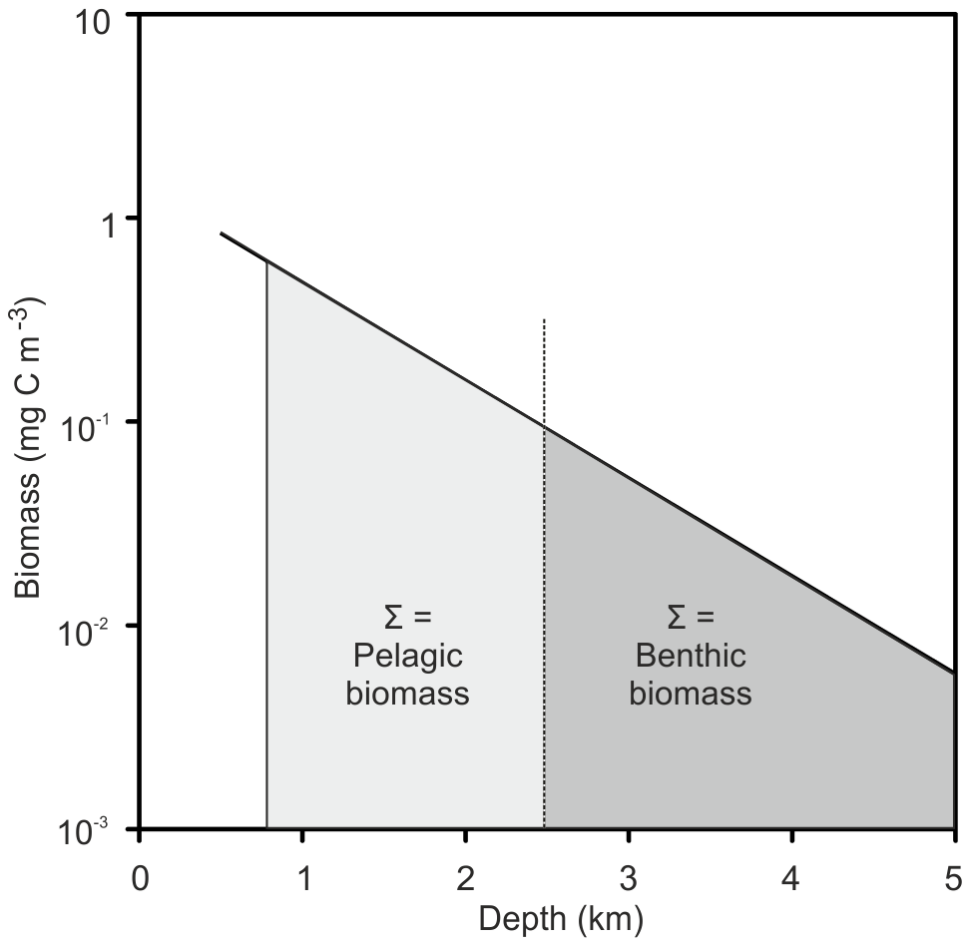
The relationship between depth and biomass. Decrease in pelagic biomass (mg C.m^−3^) as a function of depth, back-calculated from the CoML global trend for benthic biomass (sloping line). At any given depth (indicated here by a dashed vertical line), the benthic biomass (mg C.m^−2^) is represented by the dark shaded area to the right and the total pelagic biomass above the sea floor from 800 m depth (mg C.m^−2^) by the paler shading to the left. Pelagic plus benthic biomass is hence constant.

**Figure 12:**
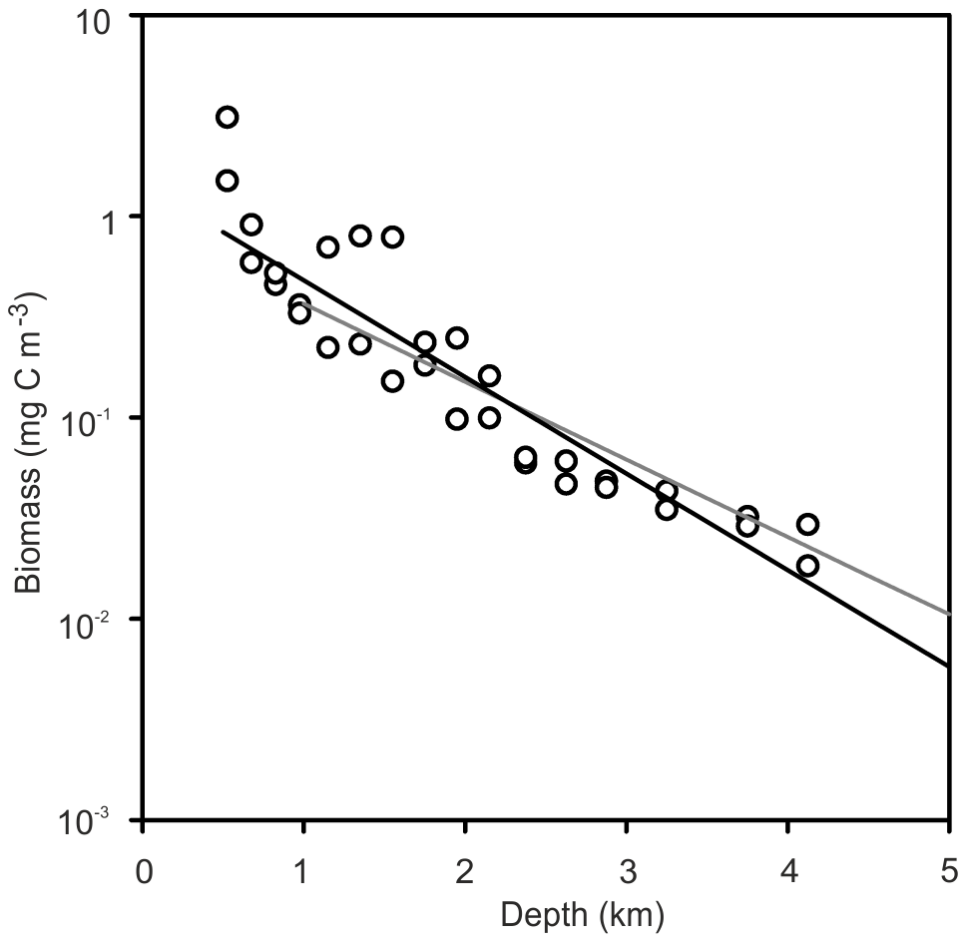
Comparison of estimates of pelagic biomass. Open circles are data from the BIOTRANS station at 47°N, 20°W east of the MAR [42]. The grey line is based on data from 31°17′N 25°24′ W over the Madeira Plain [43]. The solid black line is the theoretical relationship derived from integrating the CoML global trend for benthic biomass as shown in Figure 12.

In Figure 11 the benthic biomass is seen to correspond to the dark shaded integrated area to the right of the depth marker, i.e. equivalent to the pelagic biomass that would be present if the ocean were infinitely deep. Fitting values P_0_ = 1.45 mgC.m^−3^ and k  = 4.8×10^−4^ m^−1^ into equation (3) makes the result equal to the CoML global prediction of macrofauna plus megafauna biomass [38] (Figure 12). P_0_ is the theoretical global mean pelagic biomass at the sea surface and k the slope of decline with depth. Here we only use the equation to predict biomass at depths>800 m, away from surface patchiness and non-linear distributions of biomass associated with features such as deep scattering layers. Equation (1) therefore can be used to describe global deep pelagic and benthic biomass. Surface biomass and export flux from the surface will vary spatially and temporally with some time lags. It is beyond the scope of the present study to investigate these effects. We conclude that our observation for the MAR in the North Atlantic is applicable globally and that presence of sea mounts and mid-ocean ridges may have minimal influence on total deep sea biomass if displacement of pelagic biomass by the volume of these features is taken into account.

### Biodiversity on the MAR

**Figure 13:**
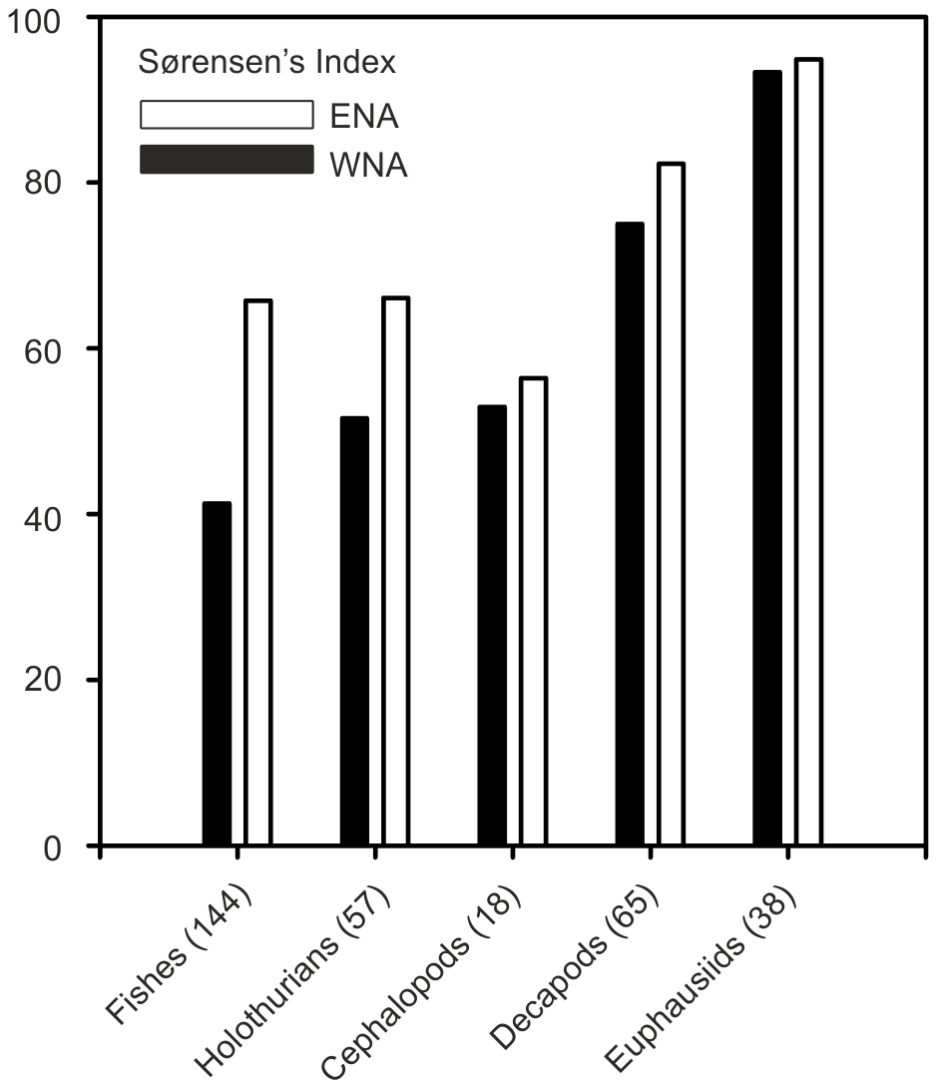
Faunal overlap between the MAR and continental slopes. Sørensen indices of species overlap between MAR and the Northwest (WNA, filled) and Northeast (ENA, open) Atlantic derived from species occurrence data. Numbers in parentheses are the numbers of species of each group found on the MAR.

We found the demersal and benthic species populating the MAR to be mainly typical North Atlantic lower bathyal species known from the ocean margins. For most taxa, endemicity is not high [see File S1]; zero for fishes [S12, S13, S14, S15, S16, S17, S18], cephalopods [S43, S43, S44, S45, S46, S47, S48, S49, S50, S51] and at least one meiofaunal taxon (benthic foraminifera) and ∼10% for benthic megafauna. Holothurians apparently exhibit higher endemism (∼ 18%) [S33, S34, S35, S36, S37, S38, S39, S40, S41, S42]. In contrast for euphausiids, which occur mainly in the epi-and upper mesopelagic zone, there was 95% species overlap between the MAR and continental margin sites [S62,S63]. Comparison of species lists from the MAR with published records for the North Atlantic margins (see File S1) shows stronger similarities with the eastern rather than the western Atlantic (Figure 13). Using similar gear, demersal fish species richness was 9.5 per trawl at the MAR stations (SD = 2.4), 14.1 (SD = 2.9) at the same depth at PSB, and 3.8 (SD = 1.2) at 4800 m depth at PAP [44], indicating that mid-ocean elevation of the sea floor does increase biodiversity compared with an abyssal plain but demersal fish species richness was equal to, or less than, values on the ocean margins at the same depths. Overall, there is no evidence that biodiversity on the MAR is greater than on comparable ocean margin areas. Species generally exhibit depth fidelity e.g. 90.3% of trawled megafauna species common to the PSB and the MAR occurred at similar depth ranges. However, some species with predominantly abyssal distribution (>3500 m depth) were found in great abundance at ∼2500 m depth at the MAR stations, e.g. *Abyssorchomene abyssorum* and *A. chevreuxi* (Amphipoda) and *Kolga nana* (Holothuroidea).

## Discussion

Our investigations of waters overlying the MAR indicate that in this region, primary production, downward export flux of organic carbon and pelagic biomass are not significantly different from values in adjacent waters overlying abyssal plains. There is no evidence for a putative ridge effect enhancing regional biological productivity. This is in contrast to oceanic islands and sea mounts in the Southern Ocean where natural iron fertilisation of surface waters does locally increase primary production, export flux and deep-sea benthic biomass [45], [46] Our acoustic surveys (Fig 5) and previous acoustic surveys across the MAR at 43°53′N and 56°N show some association of pelagic fauna with local topography [47]. Moreover, in contrast to the general exponential decline in biomass with depth in open-ocean systems [48], pelagic trawl samples revealed a deep-pelagic fish biomass maximum between 1500 and 2300 m depth along the ridge crest [49]. We hypothesise that these concentrations of biomass and biodiversity do not reflect localized increased biological productivity but is the result aggregation behavior by active nektonic species effectively importing biomass from the surrounding seas.

Benthic biomass on the MAR is enhanced compared with a hypothetical mid ocean with a continuous abyssal plain and no ridge. This is not the result of an increase in total biological productivity; displaced pelagic biomass is simply replaced by benthic biomass. We propose that generally for deep ocean areas, for depths greater than 800 m, pelagic plus benthic biomass is constant, the value of the total being determined by the mean primary production in the photic zone of the area under consideration. Our analysis did not include the smaller mieofauna fraction which also follow depth trends similar to the macrofauna and megafauna [38]. Benthic meiofauna together with smaller size fractions of plankton would contribute to the total biomass present. We did not measure biomass on hard substrata, rocky outcrops and cliffs that harbour a diverse assemblage of sessile fauna dominated by corals, sponges and crinoids [33]. However such habitat represents<5% of the lower bathyal area of the MAR. Although patches of attached fauna may be very conspicuous, indeed made more so by bioluminescence [50], a large proportion of the lower bathyal rock faces is bare (Figure 3). Our calculations imply that biomasses on hard and soft substrate are equal; any error arising from this is likely to be small.

Comparisons of biodiversity and evidence for endemism are hampered by our imperfect knowledge of deep-sea fauna. New species discovered [51], [52] on the MAR are likely to be found living elsewhere. Although ca. 18% endemism amongst holothurians appears to be well founded, the fact that the best known taxon, the fishes, shows zero endemism may be more informative. Indeed population genetic studies on some deep demersal fish species suggest that the MAR is not a barrier to gene flow; roundnose grenadier (*Coryphaenoides rupestris*)[53] showed only slight intra-specific differentiation from the ocean margins [54] and in blue hake (*Antimora rostrata*) there was no differentiation [55]. Analysis of species occurrences shows a clear bias towards closer similarity to the eastern Atlantic margin (Figure 13).

There is no doubt that presence of the MAR greatly alters the water circulation and biology of the Atlantic Ocean providing habitat for bathyal organisms that would not otherwise survive in mid ocean. However the overall effect on oceanic productivity appears to be neutral which cautions against excessive ambitions for exploitation of biological resources. From the point of view of biodiversity, the MAR roughly doubles the available area of lower bathyal habitat in the ocean basin. Applying species-area theory [56] this suggests that the MAR is more important for sustaining bathyal benthic diversity in the Atlantic basin as a whole rather than supporting a rich endemic fauna of its own.

## Supporting Information

Figure S1 **The relationship between pelagic and benthic biomass.** The curve indicates a trend of pelagic biomass density as a function of depth. For any given bottom depth (*H*) the integrated area under curve to the right is equal to the benthic biomass per unit surface area. The integrated area to the left gives the pelagic biomass per unit surface area. (tiff)
(TIF)

File S1 **Comprising information on Cruises, Materials & Methods, The relationship between pelagic and benthic faunal biomass, Biodiversity Data Sources used in Figure 13, and Supporting Information References to sources of data.**
(DOCX)
