# Supplementary figures and images for: Does Presence of a Mid-Ocean Ridge Enhance Biomass and Biodiversity?
Source: PLoS One. 2013 May 2;8(5):e61550. doi: 10.1371/journal.pone.0061550 (PMC3642170; doi:10.1371/journal.pone.0061550)

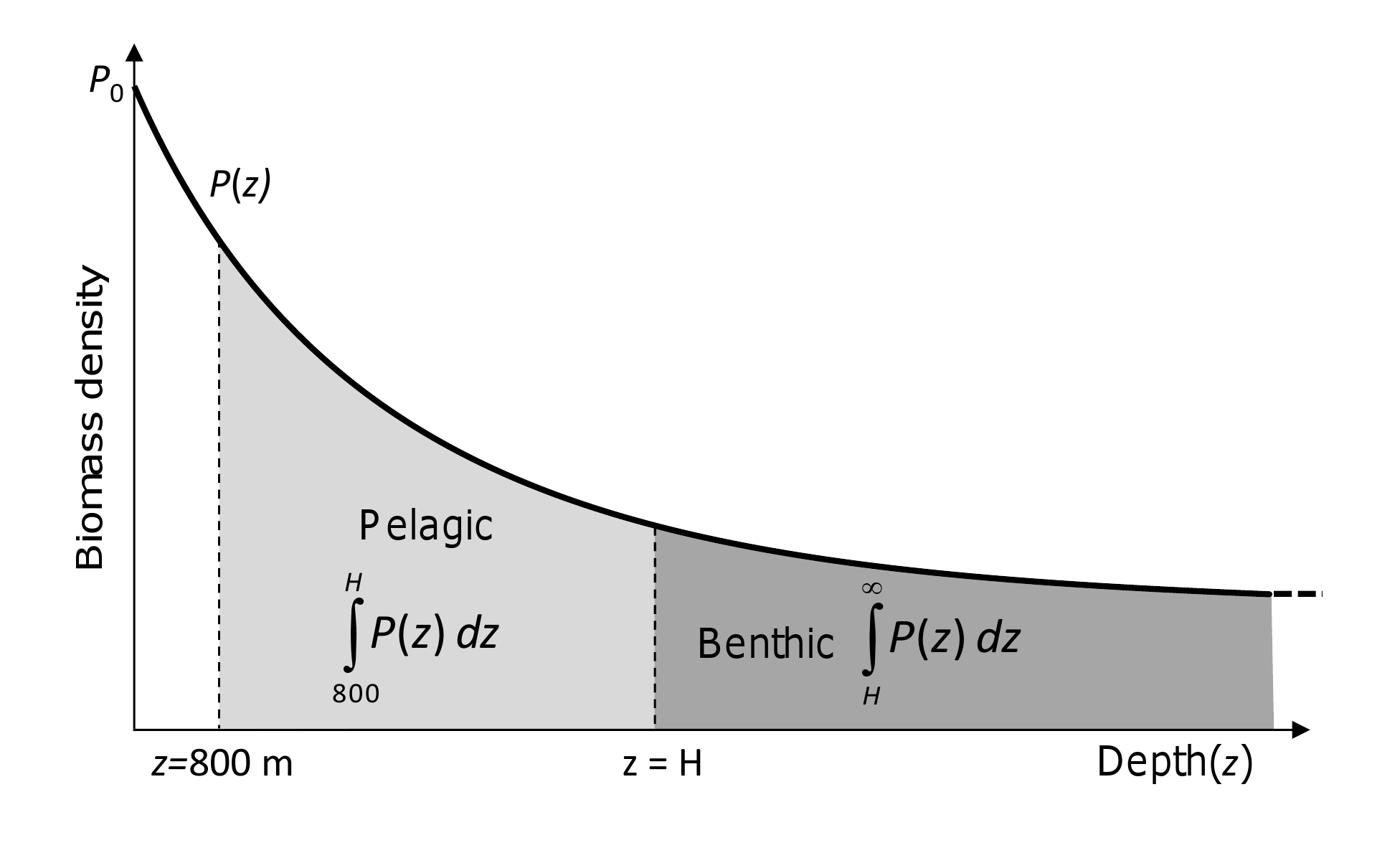

Supplement: Figure S1 — The relationship between pelagic and benthic biomass. The curve indicates a trend of pelagic biomass density as a function of depth. For any given bottom depth (H) the integrated area under curve to the right is equal to the benthic biomass per unit surface area. The integrated area to the left gives the pelagic biomass per unit surface area. (tiff) (TIF) [file pone.0061550.s001.tif]
