# Supplementary material for: Does Presence of a Mid-Ocean Ridge Enhance Biomass and Biodiversity?
Source: PLoS One. 2013 May 2;8(5):e61550. doi: 10.1371/journal.pone.0061550 (PMC3642170; doi:10.1371/journal.pone.0061550)
Supplement: File S1 — Comprising information on Cruises, Materials & Methods, The relationship between pelagic and benthic faunal biomass, Biodiversity Data Sources used in Figure 13, and Supporting Information References to sources of data. (DOCX) [file pone.0061550.s002.docx]

**Supporting Information for:**

Does presence of a Mid-Ocean Ridge enhance biomass and biodiversity?

**Imants G. Priede*, Odd Aksel Bergstad*, Peter I. Miller,**

**Michael Vecchione, Andrey Gebruk, Tone Falkenhaug, David S.M. Billett, Jessica Craig, Andrew C. Dale, Mark A. Shields, Gavin H. Tilstone,**

**Tracey T. Sutton, Andrew J. Gooday, Mark E. Inall, Daniel O.B. Jones, Victor Martinez-Vicente, Gui M. Menezes, Tomasz Niedzielski,**

**Þorsteinn Sigurðsson, Nina Rothe**, **Antonina Rogacheva, Claudia H.S. Alt, Timothy Brand, Richard Abell**, **Andrew S. Brierley, Nicola J. Cousins, Deborah Crockard, A. Rus Hoelzel, Åge Høines_,_ Tom B. Letessier,**

**Jane F. Read, Tracy Shimmield, Martin J. Cox, John K. Galbraith,**

**John D.M. Gordon, Tammy Horton, Francis Neat, Pascal Lorance**.

* To whom correspondence should be addressed e-mail: [i.g.priede@abdn.ac.uk](mailto:i.g.priede@abdn.ac.uk) (I.G.P.); [oddaksel@imr.no](mailto:oddaksel@imr.no) (O.A.B.)

**Note:** Reference numbers [N] with no prefix are in the main reference list.

Reference numbers with a prefix [SN] are in the supplementary reference list.

**Cruises**

The data for this study were derived from two related research projects:

1. MAR-ECO Patterns and processes of the ecosystems of the northern mid-Atlantic

<http://www.mar-eco.no/about> an international program involving research cruises to the MAR from Russia, Germany, the United Kingdom, USA, and Portugal. A major feature was a two month voyage of the *RV GO Sars* in 2004 during which numerous samples and data were collected [S1].

1. ECOMAR Ecosystems of the Mid-Atlantic Ridge at the sub polar front and the Charlie-Gibbs Fracture Zone. <http://www.oceanlab.abdn.ac.uk/ecomar/>

This UK-funded project contributed to MAR-ECO through the following cruises. Reports can be downloaded at <http://www.oceanlab.abdn.ac.uk/ecomar/cruises.php>

*RRS James Cook* JC011 13th July - 19th August 2007

Southampton, U.K. to Clyde, U.K.

*RRS Discovery* D331 23rd July - 18th August 2008.

Portland UK to St John's, Newfoundland, Canada.

*RRS James Cook* JC037 1 August – 9 September 2009

Southampton UK to Falmouth UK.

*RRS James Cook* with the *ROV Isis.* JC 048 2010 26 May – 3 July 2010.

St Johns, Newfoundland, Canada to Vigo, Spain.

All data from these cruises and cruise reports are archived and can be accessed from the British Oceanographic Data Centre: <http://www.bodc.ac.uk/>

**Materials and Methods**

Benthic Biomass - Direct Measurement

Macrofauna were sampled by replicate megacorer casts (8 core tubes fitted, internal core diameter 10 cm) at each of the four MAR stations (Figure 1*B*). The mean number of successful cores per cast was 6 (range 3 – 8). Wet weight of material retained on a 500 µm mesh sieve was determined [S2] and converted to organic carbon weight with appropriate conversion factors [S3]. Megafauna and fishes were collected by an otter trawl towed on a single warp [S4] from the *RRS James Cook* at the NW, NE and SE station MAR stations. The extent of flat terrain at the SW station was insufficient for trawling. These samples could be directly compared with data from PAP and PSB collected using the same gear. The effective opening width of the OTSB is about 8.6 m, and the net is composed of 43 mm and 37 mm stretch mesh netting, reducing to 13 mm stretch mesh in the cod end. Tow duration was 1 to 1.5h on the sea floor at 2-2.5 knots. The *GO Sars* in 2004 used a Campelen 1800 shrimp trawl fished on twin warps with rock hopper gear at depths 607 to 3527m [S1]. Distance towed and wing spread were used to calculate area covered. Carbon content was estimated by applying appropriate conversion factors to wet weights.

Biodiversity

In addition to the biomass sampling, collections were made using baited traps for necrophagous species, baited long lines for larger fish species (*MS Loran*) and tools carried by remotely operated vehicles. Meiofauna (Foraminifera) were retrieved from the upper 0-1cm layer of core samples.

Biomass - Sources of Comparative Data from around the North Atlantic Ocean.

1. *Benthic macrofauna biomass* (Figure 7)

Macrofaunal biomass values from the MAR were compared with similar depths on the continental margin of the eastern North Atlantic [36,37, S5] and western North Atlantic [S6,S7].

(ii) *Benthic megafauna biomass* (Figure 8)

Megafaunal density values were compared between trawl surveys on the MAR (*RRS James Cook* OTSB trawls), the Porcupine Area of the eastern North Atlantic (OTSB data from 2500 ± 100 m were selected from an original dataset [S8] supplied by B. Bett and the deep waters south of New England on the western North Atlantic (OTSB and shrimp-trawl data from the 2116-2481m and 2504-3113 m zones [S9, S10]. All biomass data were converted to organic carbon weight from wet weight using a 2% conversion factor [S11] and standardized to g C m^-2^.

(iii)  *Demersal Fish* (Figure 9)

Estimates of demersal fish biomass were derived from a number of bottom trawl studies sampling extensive depth ranges of western and eastern North Atlantic slopes [S12, S13, S14, S15, S16, S17 ], and the MAR [S18]. Additional datasets came from French surveys of the Bay of Biscay, Irish slope (P. Lorance, IFREMER) and from the slope off of New England and the middle Atlantic Bight (J. Galbraith, NOAA, NMFS) [40]. Swept-area estimates of catch rates in terms of kg.1000m^-2^ for single tows with twin-warp trawls were calculated and compared. The area swept was estimated as the distance between the wings of the trawl times the distance towed, i.e. using measures provided for each study and trawl type.

Overall biomass estimates within an area derived by different trawls are likely to be comparable [39] but standardisation by swept area may not always be sufficient to compensate for gear differences. An additional independent analysis, data from a single trawl type, i.e. the single-warp OTSB trawl, was used to compare biomass indices from the same depth, i.e. near 2500m, between the MAR (own data from *RR James Cook* cruises, 2007-2010), the Porcupine Seabight [S19], and the northwest Atlantic [S11].

Estimation of deep pelagic biomass displaced by the MAR.

For the North Atlantic, biomass of mesozooplankton at the BIOTRANS site 47° 20° W between 4250 and 2250 m depth [41] has been estimated as 0.04 mg C.m^-3^. Taking into account smaller and larger plankton and nekton size classes we assume the total biomass in water displaced by the ridge is likely to be greater. Further estimates of pelagic biomass at 2000-4000 m depth were derived from a number of published data from the North Atlantic [S21, S22, S23, S24, S25, 42] and were converted from wet to dry weight (9.3% conversion factor [36] and dry weight to organic carbon weight (60% conversion factor [S2]) giving estimates of pelagic biomass in the North Atlantic of 0.02-0.8 mg C.m^-3^. Despite differences in sampling mesh sizes, the biomass estimates from the North Atlantic investigations are surprisingly similar. An average value of 0.06 mg C.m^-3^ was employed for the calculation of the pelagic biota biomass displaced by the MAR

**The relationship between pelagic and benthic faunal biomass**

We have shown from the above calculations that the pelagic biomass displaced by the MAR is approximately equal to the enhancement in benthic biomass as predicted by the Wei *et al* equations [38]. This could be the result of a peculiarity of the shape of the MAR but we hypothesise that it is more likely due to a general principle that deep pelagic biomass plus benthic biomass per unit area is constant.

In order for this to be true, if pelagic biomass varies as a function of depth *P(z)* then in any given water depth *H* the benthic biomass per unit area *B(z)* should be equal to the integrated area under the pelagic biomass curve as shown in Figure S1:

$$B(z)=\int_{H}^{\infty} P(z)\text{ }dz$$

Since we know the relationship between benthic biomass and depth, (macrofauna plus megafauna, i.e.material retained by a 250µm sieve) from the equations provided by Wei et al. [38] we can back-calculate the relationship between pelagic biomass and depth. This is simplified by the assumption according to Roe that pelagic biomass decreases logarithmically with depth [42]:

$$P(z)=P_{0}\cdot10^{-kz}$$

By a process of iteration we have found that *P_0_* = 1.45 mgC.m^-3^ and *k* = 4.8×10^-4^ m^-1^ provides a good fit to the CoML global trends [38] for benthic biomass. Plotting this curve in Figure 12 shows a good correspondence with available pelagic biomass data for the North Atlantic Ocean giving further evidence supporting the general principle (pelagic biomass + benthic biomass = constant).

**Biodiversity Data sources** (Figure 13)

*(i) Demersal Fishes*

Presence-absence data on demersal fish species on the MAR between Iceland and the Azores [40. S26] were compared with information from the continental margins on either side of the Atlantic [ S27, S28, S29, S30, S31, S32] including data from the ECOMARGE project [www.ecomarg.net/biodiversidad](http://www.ecomarg.net/biodiversidad). A standard definition for demersal fishes was applied [S27].

*(ii) Holothurians*

Presence and absence data was compiled for holothurian species occurring on the MAR (from the Azores to Iceland) and in the western and eastern North Atlantic between depths of 1000 and 3500 m using original and published data on benthic and bentho-pelagic holothurians [S33, S34, S35, S36, S37, S38, S39, S40, S41, S42]. In the western North Atlantic we considered species from the equator to the southern border of the Baffin Sea and in the eastern North Atlantic from the equator to the Faeroe-Iceland Ridge.

The total number of species of holothurian included in the analysis was 87 of which 54 were found on the MAR. Of these, 10 species (18.5%) are endemics of the MAR. The majority of MAR species (22 species or 40.7%) occur in the western and eastern North Atlantic. There are 18 species (33.3%) recorded on the MAR and the eastern North Atlantic. There are only three species (5.6%) recorded on the MAR and the western North Atlantic. One species, *Mesothuria bifurcata,* has only been recorded on the MAR in the North Atlantic but also occurs in the Antarctic.

*(iii) Cephalopods*

Data were compiled for the occurrence of deep-demersal and benthopelagic cephalopod species in the North Atlantic from the published literature and unpublished personal observations. Data were available for comparable depths from the MAR (the Azores to the Charlie-Gibbs Fracture Zone plus Sub-Polar Front area) [S43], the western North Atlantic (NWA) [S43, S44, S45, S46, S47, S48, S49], primarily off New England and Nova Scotia, and the eastern North Atlantic (NEA) [S50, S51], primarily Porcupine Seabight and Rockall Trough. Although some literature exists for some of these cephalopod species in other areas of the North Atlantic (e.g., Iceland, Greenland, off Norway, Blake-Bahama Plateau), it only covers subsets of the taxa or does not extend consistently deep enough.

All observations included are based on specimens collected in bottom trawls at depths of ca. 1000 m or greater. Truly pelagic species that are sometimes caught in bottom trawls were not included in the comparisons, nor were the many species collected only by pelagic sampling. However, some species of squids and cirrate octopods that are pelagic but strongly bottom associated were included. Cirroteuthids, stauroteuthids, and mastigoteuthids are occassionally encountered far above the bottom, but commonly drift just above the bottom, presumably to feed in the enriched zooplankton of the benthopelagic layer. Some ommastrephids (e.g., *Illex illecebrosus* and presumably *Todarodes sagittatus*) sit on the bottom, even in very deep water, during the day.

*(iv) Decapods*

Basin-wide comparison of the occurrence of decapod shrimps in the northern North Atlantic include presence-absence data on decapod shrimps caught in pelagic and demersal trawls. The areas compared were 1) the MAR (40 – 60ºN), 2) the eastern North Atlantic, and 3) the western North Atlantic. The species list from the MAR was obtained from the *GO Sars* (2004) expedition and the species lists from the eastern and western North Atlantic were compiled from the published literature [S52, S53, S54, S55, S56, S57, S58, S59, S60, S61].

*(v) Euphausiids*

Pelagic Euphausiid species distribution data were collected using maps of area of occurrence [S62] overlayed on a published grid framework which considered the influence of the ridge and the alignment of the fracture zones [S63].

**Supporting Information References**

(With S prefix in the text)

S1. Wenneck TdeL, Falkenhaug T, Bergstad OA (2008) Strategies, methods, and technologies adopted on the R.V. G.O. Sars MAR-ECO expedition to the Mid-Atlantic Ridge in 2004. Deep Sea Res Part 2 Top Stud Oceanogr 55: 6–28.

S2. Gage JD, Hughes DJ, Gonzalez-Vecino JL (2002) Sieve size influence in estimating biomass, abundance and diversity in samples of deep-sea macrobenthos. Mar Ecol Prog Ser 225:97–107.

S3. Rowe GT (1983) in *Deep-Sea Biology* ed Rowe GT (Wiley, New York) pp 97–121.

S4. Gordon JDM, Bergstad OA, (1992) Species composition of demersal fish in the Rockall Trough, north- eastern Atlantic, as determined by different trawls. J Mar Biol Assoc U.K. 72:213-230.

S5. Pfannkuche O, Soltwedel T (1998) Small benthic size classes along the N.W. European Continental Margin: spatial and temporal variability in activity and biomass. Prog Oceanogr 42:189-297.

S6. Rowe GT, Polloni PT, Horner SG (1974) Benthic biomass estimates from the northwestern Atlantic Ocean and the northern Gulf of Mexico. Deep Sea Res Part 1 Oceanogr Res Pap 21: 641-650.

S7. Rowe et al. GT (2008) Comparative biomass structure and estimated carbon flow in food webs in the deep Gulf of Mexico. Deep Sea Res Part 2 Top Stud Oceanogr 55: 2699-2711.

S8. Thurston MH, Bett BJ, Rice AL, Jackson PAB (1994) Variations in the invertebrate abyssal megafauna in the North Atlantic Ocean. Deep Sea Res Part 1 Oceanogr Res *Pap* 41: 1321-1348.

S9. Haedrich RL, Rowe GT, Polloni T (1980) The megabenthic fauna in the deep-sea south of New-England, USA. Mar Biol 57:165-179

S10. Lampitt RS, Billett DSM, Rice AL (1986) Biomass of the invertebrate megabenthos from 500 to 4100 m in the Northeast Atlantic Ocean. Mar Biol 93: 69-81.

S11. Haedrich RL, Rowe GT(1977) Megafaunal biomass in the deep sea. Nature 269: 141-142.

S12. Gordon JDM, Bergstad OA, (1992) Species composition of demersal fish in the Rockall Trough, north- eastern Atlantic, as determined by different trawls. J Mar Biol Assoc U.K. 72:213-230

S13. Bailey DM, Collins MA, Gordon JDM, Zuur AF, Priede IG (2009) Long-term changes in deep-water fish populations in the North East Atlantic: deeper-reaching effect of fisheries? Proc R Soc Lond B Biol Sci 275: 1965-1969.

S14. Gordon JDM, Duncan J (1985) The ecology of the deep-sea benthic and benthopelagic fish of the slopes of the Rockall Trough, Northeastern Atlantic. Prog Oceanogr 15: 37-69.

S15. Gordon JDM (1986) The fish populations of the Rockall Trough. Proc R Soc Edinb Biol 88B: 191-204.

S16. Snelgrove PRV, Haedrich RL (1985) Structure of the deep demersal fish fauna off Newfoundland. Mar Ecol Prog Ser 27: 99-107.

S17. Neat F, Burns F (2010) Stable abundance, but changing size structure in grenadier fishes (Macrouridae) over a decade (1998–2008) in which deepwater fisheries became regulated. Deep Sea Res Part 1 Oceanogr Res Pap I 57: 434-440.

S18. Bergstad OA, Menezes G, Høines ÅS (2008) Distribution patterns and structuring factors of deepwater demersal fishes on a mid-ocean ridge. Deep Sea Res Part 2 Top Stud Oceanogr 55: 185-202.

S19. Merrett NR, Haedrich RL, Gordon JDM, Stehmann M (1991) Deep demersal fish assemblage structure in the Porcupine Seabight (eastern North Atlantic): results of single warp trawling at lower slope to abyssal soundings. J Mar Biol Assoc U.K., 71: 359-373.

S20. Haedrich RL, Rowe GT(1977) Megafaunal biomass in the deep sea. Nature 269: 141-142.

S21. Jaschnov VA (1961).Vertical distribution of the mass of zooplankton in the tropical region of the Atlantic Ocean. Oceanology 136-141: 4-6.

S22. Grice GD, Hulsemann K (1965) Abundance, vertical distribution and taxonomy of calanoid copepods at selected stations in the northeast Atlantic. J Zool 146:213-262.

S23. Wishner KF (1965) The biomass of the deep-sea benthopelagic plankton. Deep Sea Res A 27: 203-216.

S24. Angel MV, Baker A deC (1982) Vertical distribution of the standing crop of plankton and micronekton at three stations in the northeast Atlantic. Biol Oceanogr 2: 1-30.

S25. Beckmann W (1988) The zooplankton community in the deep bathyal and abyssal zones of the eastern North Atlantic. Preliminary results and data lists from MOCNESS hauls during cruise 08 of the RV 'POLARSTERN'. Ber. Polarforsch. 42: 1-57.

S26. Hareide N-R, Garnes G (2001) The distribution and catch rates of deep water fish along the Mid-Atlantic Ridge from 43° to 61° N. Fish Res 51:297-310.

S27. Haedrich RL, Merrett NR (1998) Summary atlas of deep-living fishes in the North Atlantic. J Nat Hist 22:1325-1362.

S28. Magnussen E (2002) Demersal fish assemblages of Faroe Bank: species composition, distribution, biomass spectrum and diversity. Mar Ecol Prog Ser 238: 211-225.

S29. Moore JA, Hartel KE, Craddock JE, Galbraith JK (2003) An annotated list of deepwater fishes from off the New England region, with new area records. Northeast Nat 10:159-248.

S30. Musick JA, Desfosse JC, Grogan E (1992) Fish assemblages at Deepwater sewage disposal site (DWD-106). Final Report submitted to National Marine Fisheries Service. 60p.

S31. Musick JA, Desfosse JC, Wilk S, McMillan D, Grogan E (1996) Historical comparison of the structure of demersal fish communities near a deep-sea disposal site in the western North Atlantic. Journal of Marine Environmental Engineering 3: 149-171.

S32. Hartel KE, Craddock JE (2002) in *Bigelow and Schroeder's Fishes of the Gulf of Maine* 3rd Ed. eds. Collette BB, Klein-MacPhee G. Washington, DC, Smithsonian Press pp191-194.

S33. Deichmann E (1930) The holothurians of the western part of the Atlantic Ocean. Bull Mus Comp Zool 71:41-226.

S34. Gage JD, Billett DSM, Jensen M, Tyler PA (1985) Echinoderms of the Rockall Trough and adjacent areas. 2. Echinoidea and Holothuroidea. Bulletin of the British Museum (Natural History). Series Zoology 48:173-213.

S35. Gebruk A (2008) Holothurians (Holothuroidea, Echinodermata) of the northern Mid-Atlantic Ridge collected by the G.O. Sars MAR-ECO expedition with descriptions of four new species. Mar Biol Res 4:48-60.

S36. Harvey R, Gage JD, Billett DSM, Clark AM, Paterson GLJ (1988) Echinoderms of the Rockall trough and adjacent areas 3. Additional records. Bulletin of the British Museum (Natural History). Series Zoology 54: 153-198.

S37. Heding SG (1942) Holothurioidea. II. Aspidochirota - Elasipoda - Dendrochirota. The Danish Ingolf Expedition IV:1-39.

S38. Herouard E (1923) Holothuries provenant des campagnes des yachts "Princesse-Alice" et "Hirondelle II" (1898-1915). Resultats des Campagnes Scientifiques du Prince de Monaco 66 : 1-163.

S39. Madsen FJ (1953) Holothurioidea. Reports of the Swedish Deep-sea Expedition, Zoology 2: 149-173.

S40. Pawson D, Vance D, Ahearn C (2001) Western Atlantic sea cucumbers of the Order Molpadiida (Echinodermata: Holothuroidea). Bulletin of the Biological Society of Washington **10**, 311-327.

S41.Perrier R (1902) Holothuries. Expeditions scientifiques Travailleur et du Talisman 273-554.

S42. Sibuet M (1977) Repartition et diversite des Echinodermes (Holothurides - Asterides) en zone profonde dans le Golfe de Gascogne. Deep Sea Res A. 24: 549-563.

S43. Vecchione M, Young RE, Piatkowski U (2010) Cephalopods of the northern Mid-Atlantic Ridge. Mar Biol Res 6: 25-52.

S44. Collins MA, Villanueva R (2007) Taxonomy, ecology and behaviour of the cirrate octopods. Oceanogr Mar Biol Ann Rev. 44: 277-332.

S45. Moore JA, Vecchione M, Collette BB, Gibbons R, Hartel KE, et al. (2003) Biodiversity of Bear Seamount, New England Seamount Chain: Results of exploratory trawling. J Northw Atl Fish Sci 31: 363-372.

S46. Moore JA, Vecchione M, Collette BB, Gibbons R, Hartel KE (2004) Selected fauna of Bear Seamount (New England Seamount Chain), and the presence of "natural invader" species. Arch Fish Mar Res 51(1-3): 241-250.

S47. Vecchione M (2001) in Island in the Stream. Oceanography and Fisheries of the Charleston Bump, American Fisheries Society Symposium 25: 153-160.

S48. Vecchione M, Galbraith J (2001) Cephalopod species captured by deepwater exploratory fishing off New England. Fish Res 51: 385-391.

S49. Vecchione M, Pohle G (2002) Midwater cephalopods in the western North Atlantic Ocean off Nova Scotia. Bull Mar Sci 71: 883-892.

S50. Allcock AL, Strugnell JM, Ruggiero H, Collins MA, (2006) Redescription of the deep-sea octopod *Benthoctopus normani* (Massy 1907) and a description of a new species from the Northeast Atlantic. Mar Biol Res 2: 372-387.

S51. Collins MA, Yau C, Allcock L, Thurston MH (2001) Distribution of deep-water benthic and benthopelagic cephalopods from the north-east Atlantic. J Mar Biol Assoc U.K*.* 81: 105-117.

S52. Vereshchaka AL, (2009) Revision of the genus Sergestes (Decapoda, dendrobranchiata, Sergestidae): taxonomy and distribution. Galathea Reports 22:7-140.

S53. Chace FA (1947) The deep-sea prawns of the family Oplophoridae in the Bingham Oceanographic collection. Bulletin of the Bingham Oceanographic Collection 11: 1-51.

S54. Crosnier A, Forest J (1973) Les crevettes profondes de l'Atlantique oriental tropical. Faune tropicale ORSTOM 19:1-409.

S55. Domanski P (1986) The near-bottom shrimp faunas (Decapoda: Natantia) at two abyssal sites in the Northeast Atlantic Ocean. Mar Biol 93: 171-180.

S56. Hargreaves PM (1984) The distribution of Decapoda (Crustacea) in the open ocean and near-bottom over an adjacent slope in the northern North-east Atlantic Ocean during autumn 1979. J Mar Biol Assoc U.K. 64:829-857.

S57. Sivertsen E, Holthuis LB (1956) Crustacea Decapoda (the Penaeidea and Stenopodidea excepted). Report on the Scientific Results of the “Michael Sars” North Atlantic deep-sea Expedition*.* 5: 3-54.

S58. Squires HJ (1966) Distribution of decapod Crustacea in the Northwest Atlantic. Am. Geogr. Soc. Serial Atlas of Marine Environ. 12: 4 p, 4 pls

S59. Sund O (1920), Peneides and Stenopides from the ”Michael Sars” North Atlantic Deep-Sea Expedition 1910. Report on the Scientific Results of the “Michael Sars” North Atlantic deep-sea Expedition. 3(7):1-36, pls.1-2 .

S60. Vereshchaka AL (1994) North Atlantic and Carribean species of *Sergia* (Crustacea, decapoda, sergestidae) and their horizontal and vertical distribution. Steenstrupia 20: 73-95.

S61. Vereshchaka AL, (2000) Revision of the genus *Sergia* (Decapoda, dendrobranchiata, Sergestidae): taxonomy and distribution. Galathea Reports 18: 69-207.

S62. Baker CdeA, Boden BP, Brinton E (1990) A Practical Guide to the Euphausiids of the World*.* London, Natural History Museum Publications 96p

S63. Letessier TB, Cox MJ, Brierley AS (2009) Drivers of euphausiid species abundance and numerical abundance in the Atlantic Ocean. Mar Biol 156:2539-2553.
